# Exploring the role of Xingren on COVID‐19 based on network pharmacology and molecular docking

**DOI:** 10.1111/jfbc.14363

**Published:** 2022-08-07

**Authors:** Maoru Wang, Bin Yu, Jisheng Wang, Yu Wang, Libo Liang

**Affiliations:** ^1^ Drug Dispensing Department The Third Hospital of Mianyang, Sichuan Mental Health Center Mianyang China; ^2^ Department of Pharmacy, Mianyang Central Hospital School of Medicine, University of Electronic Science and Technology of China Mianyang China

**Keywords:** COVID‐19, molecular docking, network pharmacology, Xingren

## Abstract

Since the outbreak of novel Coronavirus Pneumonia 2019 (COVID‐19), the role of Almonds (Xingren) in the protection and treatment of COVID‐19 is not clear. Network pharmacology and molecular docking were used to explore the potential mechanism and potential key targets of Xingren on COVID‐19. A total of nine common targets between them were obtained, and these targets were involved in multiple related processes of GO and KEGG pathway enrichment analysis. Molecular docking showed that licochalcone B has the best binding energy (−9.33 kJ·mol^−1^) to PTGS2. They are maybe the important ingredient and key potential target. Its possible mechanism is to intervene anxiety disorder in the process of disease development, such as regulation of blood pressure, reactive oxygen species metabolic process, leishmaniasis peroxisome, and IL‐17 signaling pathway.

**Practical applications:**

Xingren is a traditional Chinese medicine that has been used and developed in China for many years. It contains a variety of active ingredients and also has the functions of relieving cough, relieving asthma, enhancing human immunity, delaying aging, regulating blood lipids, nourishing brain, and improving intelligence. In this article, the possible mechanisms of action and important targets of Xingren in the prevention and treatment of COVID‐19 were discussed through network pharmacology and molecular docking. We also found that active ingredient licochalcone B and the potential target PTGS2 are worthy of further research and analysis. At the same time, the study also provides a theoretical basis and reference for the prevention and treatment of COVID‐19 and the development of new drugs.

## Graphical abstract

A total of 9 common targets were obtained between Xingren and COVID‐19. These targets were involved in multiple related processes of GO and KEGG pathway enrichment analysis. Molecular docking showed that licochalcone B has the best binding energy (−9.33 kJ·mol^−1^) to PTGS2. They might be the essential component and possible target. Its proposed mechanism is to intervene in disease development processes such as blood pressure control, reactive oxygen species metabolic process, leishmaniasis peroxisome and IL‐17 signaling pathway.

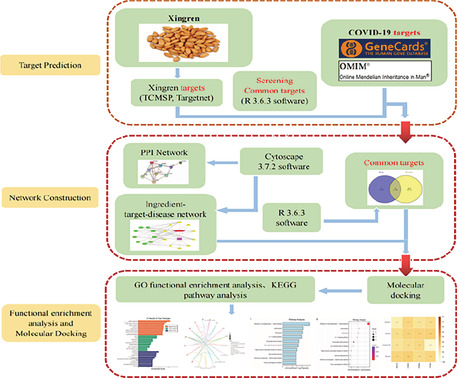

## 1 INTRODUCTION

In late December 2019, an outbreak of novel coronavirus pneumonia 2019 (COVID‐19) caused by the transmission of a new coronavirus was reported in Wuhan, Hubei Province, and became prevalent nationwide. Current studies have shown that the new coronavirus can be transmitted through respiratory droplets, close contact, contact with objects contaminated with the virus, and via aerosols in relatively closed environments, the incubation period of the virus is 1–14 days, and patients can be of all ages, and the main clinical manifestations are fever, dry cough, and malaise (Kang et al., 2022; Ni et al., 2020). Since China has a long history of Chinese medicine research and rich experience in epidemic prevention and control, Chinese medicine will play a great role in the fight against epidemics in the absence of specific drugs and vaccines. Currently, the National Health Commission of the People's Republic of China has published the “New Coronavirus Pneumonia Treatment Protocol,” which also includes Chinese medicine, and continues from the “Third Edition” to the “Ninth Edition,” and has certain efficacy in patients with mild, normal or severe COVID‐19 infection (Ding & Bian, 2020; Fang et al., 2022; Lin & Li, 2020). According to their researches, it was found that among the various ingredient preparations of traditional Chinese medicine, Almond (Xingren) is used most frequently in the therapeutic period and can be used in combination with some Chinese medicines for prescriptions (Yue et al., 2020; Zhou et al., 2020). In the treatment of COVID‐19, Xingren is also the most frequently used Chinese medicine in combination, and it is not clear whether it is used as a “ruler” medicine.

**FIGURE 1:**
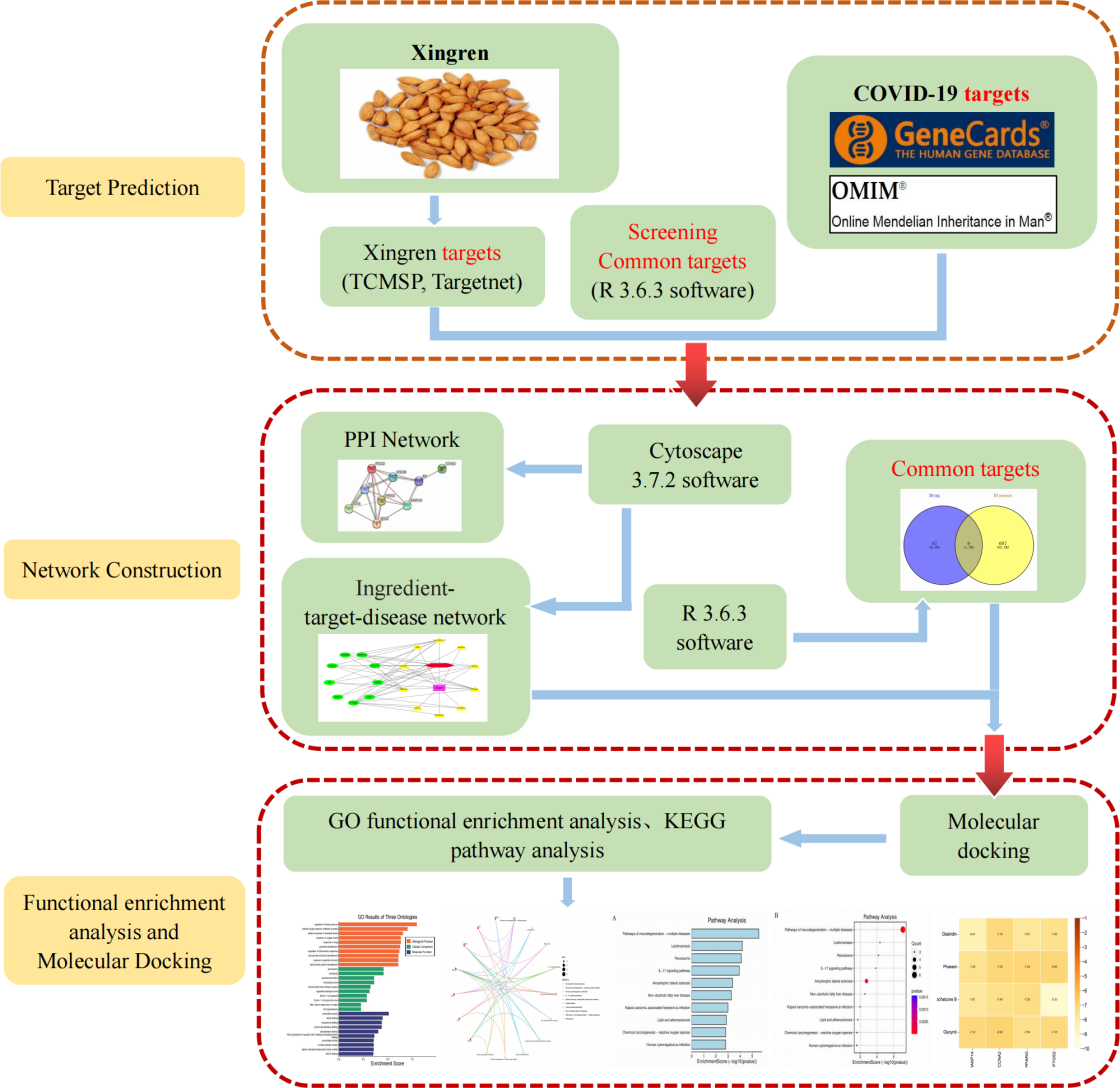
Flow chart of this study

Xingren is the seeds of the deciduous tree plant apricot (*Prunus armeniaca* L.) or bilberry, family Rosaceae, it has the effect of relieving cough and asthma and laxative and is useful for the respiratory system, digestive system, inflammatory reaction, and tumor treatment (Zheng & Shen, 2020). There are few studies on this drug in China and abroad, none of which involve studies on COVID‐19 treatment. In view of the new findings and researches for Xingren on COVID‐19, Xingren has an effect on COVID‐19, but its mechanism and core active ingredients on it are still unclear (Yue et al., 2020; Zeng et al., 2020; Zhou et al., 2020). Consequently, our team used the current emerging network pharmacology and molecular docking to investigate the material basis and potential mechanism of action of Xingren in interfering with COVID‐19 through a comprehensive analysis of the effective active ingredients, action targets and related pathways of Xingren by biological systems (Yu et al., 2022). Meanwhile, to provide a certain theoretical basis and reference for the screening of active ingredients, prevention and treatment of COVID‐19 and new drug research of Xingren by preliminary validation of molecular docking methods. The study flow is shown in Figure 1.

## 2 MATERIALS AND METHODS

### 2.1 Screening of active ingredients and action targets of Xingren

The TCMSP platform (http://tcmspw.com/tcmsp.php) was used to search for “Xingren” as the keyword, and the retrieved ingredients were screened for active ingredients with oral bioavailability (OB) > 30% and drug‐likeness (DL) > 0.18, and finally the ingredient s that met the criteria were The active ingredients were finally obtained. The active ingredients obtained from the screening were predicted using Related Targets in the TCMSP platform for the active ingredient‐related targets.

### 2.2 Collection of disease targets

The GeneCards (https://www.genecards.org/) and OMIM (https://omim.org/) platforms was searched by the keyword “novel coronavirus pneumonia”, and the relevant targets of COVID‐19 were obtained. To exclude targets with low relevance to COVID‐19, we scored according to the relevant scores of GeneCards, and we included targets with a score of ≥2. After that, we merged the two databases and deduplicated to obtain the relevant targets of COVID‐19. By taking the intersection of Xingren active ingredient‐related targets and COVID‐19‐related targets, the target that was re‐summed was the common action target of Xingren active ingredient and COVID‐19.

### 2.3 Construction of protein interaction network and active ingredient‐disease‐target network

To explore the interaction between target proteins, the common target of Xingren and COVID‐19 was uploaded to STRING (https://string‐db.org/) platform, and the species screening was set to “Homo sapiens,” and no combined score screening was performed to obtain the protein interaction PPI network. The related targets' information of Xingren and COVID‐19 was imported into Cytoscape 3.7.2 software to construct the active ingredient‐disease‐target network.

### 2.4 GO function and KEGG pathway enrichment analysis

We used R 3.6.3 software to import the coding genes of Xingren core targets into it, and executed the Cludterirofiler and pathview commands of the Bioconductor bioinformatics software package. Gene Ontology (GO) functional enrichment analysis and Kyoto Encyclopedia of Genes and Genomes (KEGG) pathway enrichment analysis were performed at *p* < .05, and the related histograms, pathway cnetplot, and bubble diagrams were obtained.

### 2.5 Molecular docking analysis

#### 2.5.1 Preparation of small molecule structures

The molecular structures of major four targets for this docking were obtained from the PubChem database (https://pubchem.ncbi.nlm.nih.gov/), and Chem3D was used for format conversion as well as energy minimization, after which all structures were imported into Schrodinger software to create a database, which was saved as a database of ligand molecules for molecular docking through hydrogenation, structure optimization, and energy minimization.

#### 2.5.2 Preparation of target protein structures

The structures of major target proteins were obtained from the RCSB database (https://www.rcsb.org/). The protein structures were processed on the Maestro 11.9 platform, and the proteins were treated with Schrodinger's Protein Preparation Wizard to remove crystalline water, fill in missing hydrogen atoms, and repair missing bond information, repair missing peptides, and finally optimize the protein for energy minimization as well as geometric structure (Fazi et al., 2015; Rajeswari et al., 2014).

#### 2.5.3 Molecular docking

Molecular docking is done by the Glide module in the Schrödinger Maestro software. Preprocessing, optimization and minimization of the receptor (constrained minimization using the OPLS3e force field). All molecules were prepared according to the default settings of the LigPrep module. When screening in the Glide module, import the prepared receptors to specify the appropriate location in the receptor grid generation. The natural ligand of the protein was selected as the center of mass for the 10 Å box. Finally, molecular docking and screening were performed by standard docking methods. The binding energy of the two bindings is expressed in kJ·mol^−1^, and the lower binding energy indicates the better binding of the two.

### 2.6 Docking results screening and analysis

The mode of interaction between the compound and the target protein was analyzed to obtain the interaction of the compound with the protein residues, such as the resulting hydrogen bonding interaction, *π*–*π* interaction, hydrophobic interaction, etc. The docking score of the compound was then referred to infer whether the compound to be screened has certain active effects. Besides, the R software was used to draw a heat map based on the molecular docking results to analyze which active ingredients were more relevant to the target.

## 3 RESULT

### 3.1 Screening results of active ingredients and action targets of Xingren

A total of 113 ingredients were retrieved from Xingren on the TCMSP platform, and 19 active ingredients and 74 drug action targets were obtained after screening, and the results are shown in Table 1.

**TABLE 1:** The active ingredients and action targets of Xingren

Mol ID	Mol name	Related targets	OB%	DL	MW
MOL010921	Estrone	CHRM3, CHRM1, CHRM5, PTGS2, CHRM4, RXRA, OPRD1, ACHE, SLC6A2, ADRA1A, PGR, CHRM2, OPRK1, SLC6A3, ADRB2, ADRA1D, SLC6A4, DRD2, OPRM1	53.56	0.32	270.4
MOL010922	Diisooctyl succinate	/	31.62	0.23	342.58
MOL002211	11,14‐eicosadienoic acid	NCOA2	39.99	0.2	308.56
MOL002372	(6Z,10E,14E,18E)‐2,6,10,15,19,23‐hexamethyltetracosa‐2,6,10,14,18,22‐hexaene	/	33.55	0.42	410.8
MOL000359	Sitosterol	PGR, NCOA2, NR3C2	36.91	0.75	414.79
MOL000449	Stigmasterol	PGR, NR3C2, NCOA2, ADH1C, RXRA, NCOA1, PTGS1, PTGS2, ADRA2A, SLC6A2, SLC6A3, ADRB2, AKR1B1, PLAU, LTA4H, MAOB, MAOA, CTRB1, CHRM3, CHRM1, ADRB1, SCN5A, ADRA1A, CHRM2, ADRA1B, GABRA1	43.83	0.76	412.77
MOL005030	Gondoic acid	PTGS1, NCOA2	30.7	0.2	310.58
MOL000953	CLR	PGR, NR3C2, NCOA2	37.87	0.68	386.73
MOL000211	Mairin	PGR	55.38	0.78	456.78
MOL000492	(+)‐catechin	PTGS1, ESR1, PTGS2, NCOA2, RXRA, CAT, HAS2	54.83	0.24	290.29
MOL002311	Glycyrol	NOS2, ESR1, PPARG, PTGS2, KDR, MAPK14, GSK3B, CHEK1, CCNA2	90.78	0.67	366.39
MOL003410	Ziziphin_qt	/	66.95	0.62	472.78
MOL004355	Spinasterol	PGR, NR3C2, NCOA2	42.98	0.76	412.77
MOL004841	Licochalcone B	NOS2, PTGS1, ESR1, AR, PPARG, PTGS2, ADRB2, ESR2, MAPK14, GSK3B, CHEK1, CCNA2	76.76	0.19	286.3
MOL004903	Liquiritin	F7, PTGS2, KDR, SOD1	65.69	0.74	418.43
MOL004908	Glabridin	NOS2, CHRM1, ESR1, AR, SCN5A, PPARG, PTGS2, RXRA, ACHE, ADRA1B, ADRB2, ESR2, MAPK14, GSK3B, CHEK1, RXRB, PRSS1, CCNA2, NCOA2, NCOA1	53.25	0.47	324.4
MOL005017	Phaseol	ESR1, AR, PPARG, PTGS2, KDR, MAPK14, GSK3B, CHEK1, CCNA2	78.77	0.58	336.36
MOL007207	Machiline	PTGS1, CHRM3, CHRM1, ADRB1, SCN5A, PTGS2, ADRA2A, ADRA2C, CHRM4, RXRA, ADRA1A, ADRA2B, ADRA1B, SLC6A3, ADRB2, ADRA1D, SLC6A4, NCOA2	79.64	0.24	285.37
MOL012922	l‐SPD	PTGS1, CHRM3, KCNH2, CHRM1, DRD5, SCN5A, CHRM5, PTGS2, ADRA2C, CHRM4, RXRA, OPRD1, ADRA1A, CHRM2, ADRA2B, ADRA1B, DRD3, SLC6A3, ADRB2, ADRA1D, SLC6A4, OPRM1	87.35	0.54	327.41

### 3.2 Results of disease target collection

**FIGURE 2:**
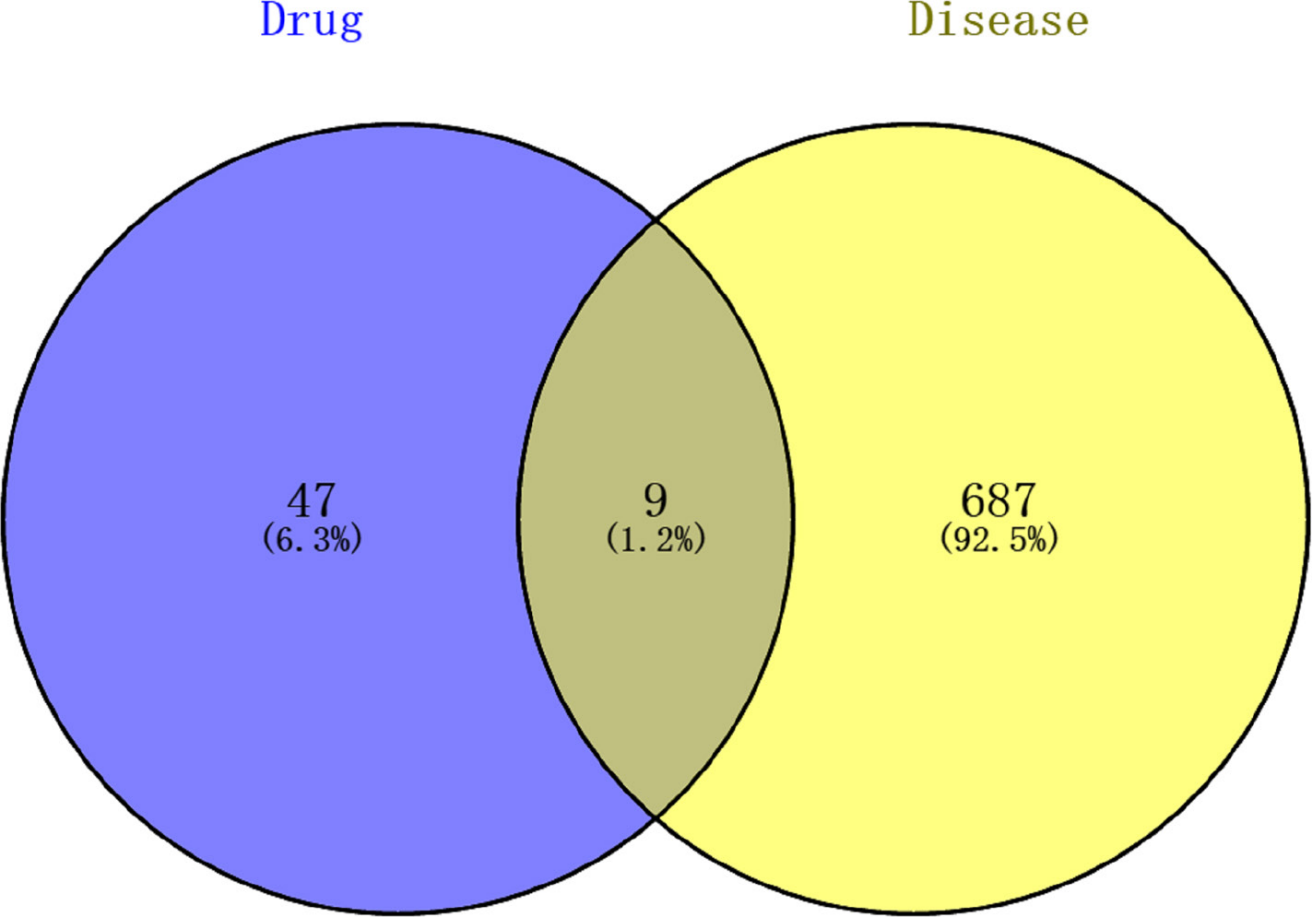
Venn diagram for common targets between Xingren and COVID‐19

A search of the GeneCards and OMIM database for relevant targets of COVID‐19 yielded 698 targets, which were filtered and deduplicated. The regulatory targets of the active ingredients of Xingren were intersected with the relevant targets of COVID‐19 using R 3.6.3 software to obtain nine common targets and the Venn diagrams of the relevant targets, and the results are shown in Figure 2.

### 3.3 Construction of protein interaction network and active ingredient‐disease‐target network

**FIGURE 3:**
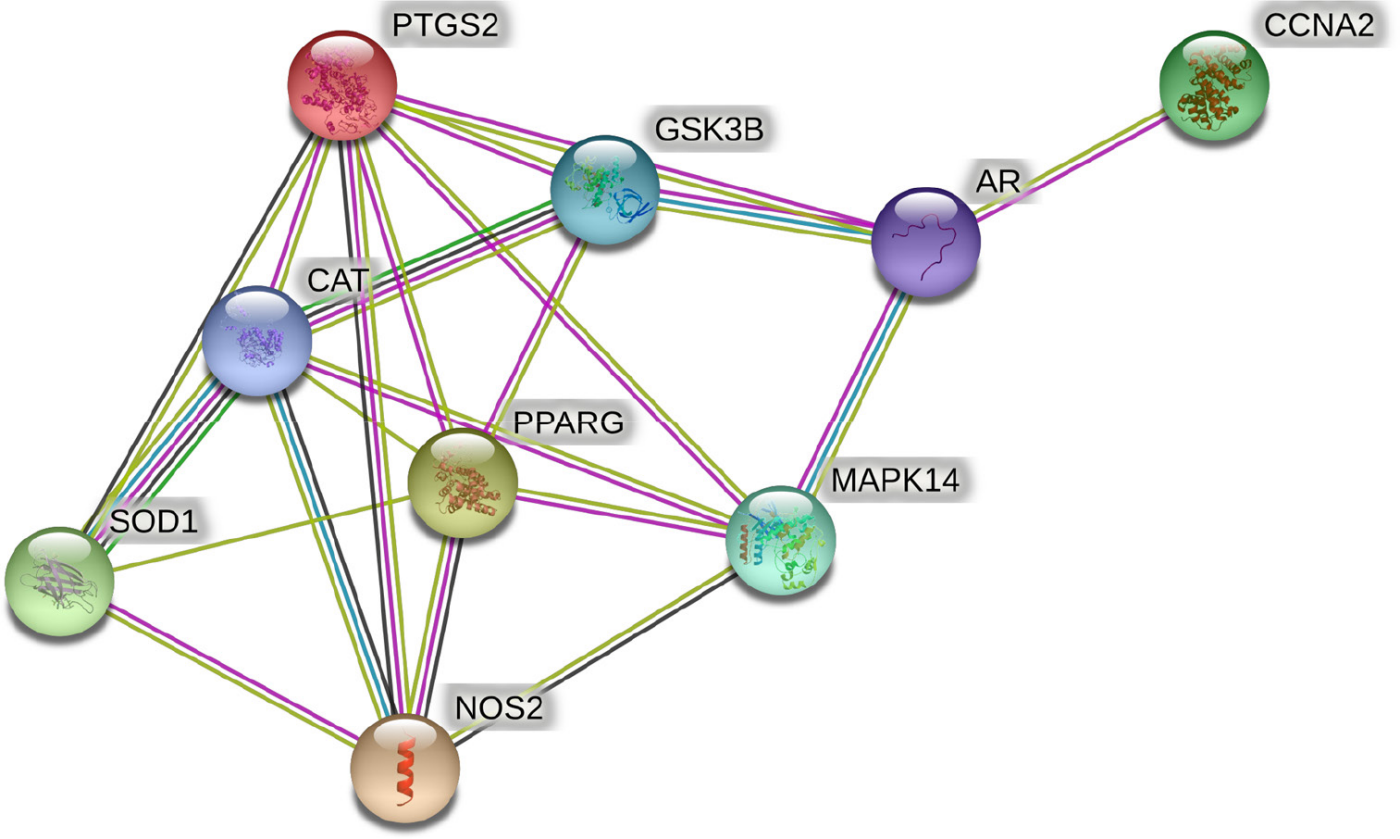
Protein interaction network of common targets of Xingren and COVID‐19

**FIGURE 4:**
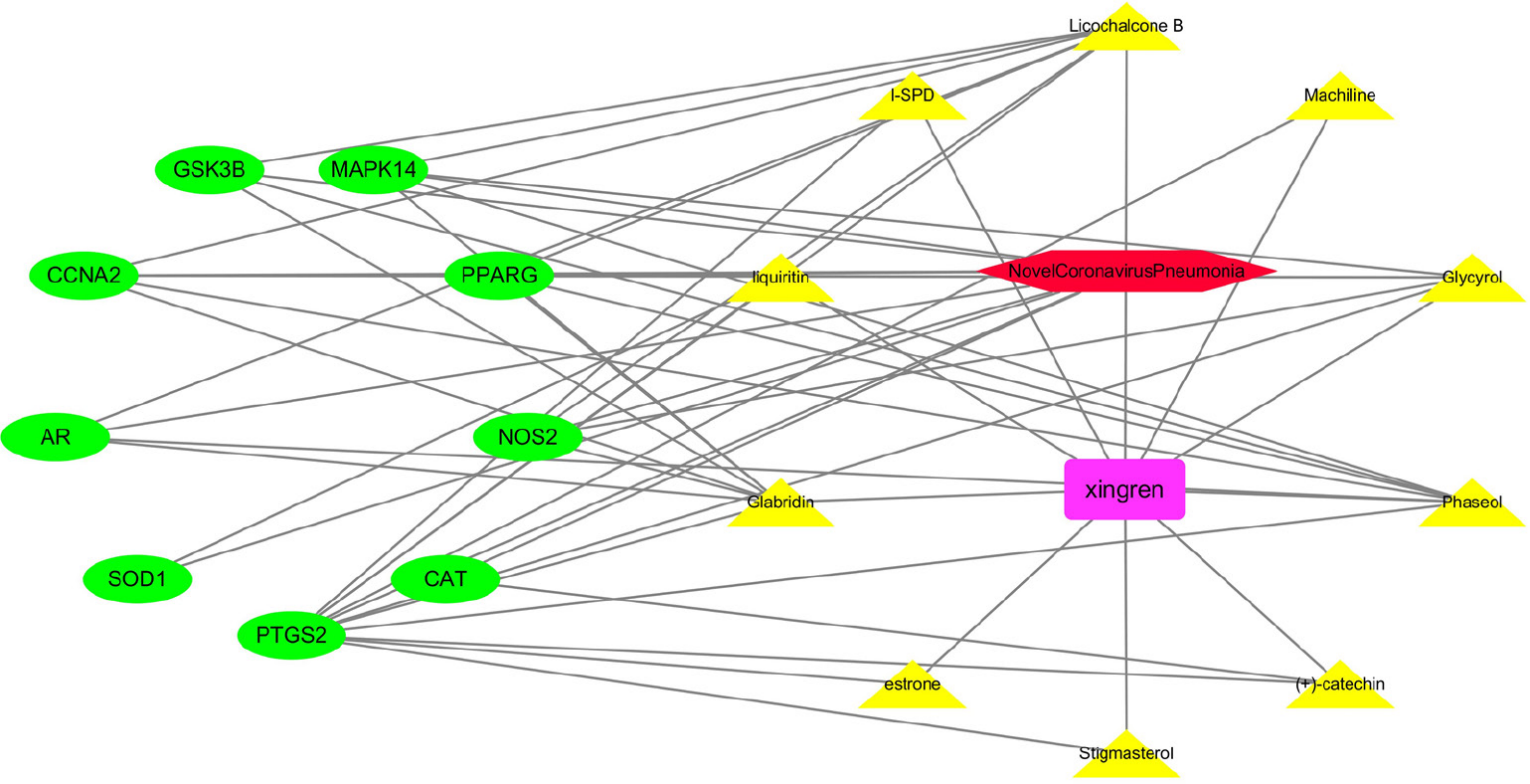
The active ingredient‐disease‐target network of Xingren on COVID‐19

By uploading the co‐interaction target of Xingren and COVID‐19 to the STRING (https://string‐db.org/) platform, and setting the species filter to “Homo sapiens” without the combined score filter, we obtained the protein Interacting PPI networks and related information, and the results are shown in Figure 3. This included 9 protein nodes, 21 interaction links, and a PPI‐enriched *p*‐value of 1.39e^−07^ (*p* < .05). The construction of active ingredient‐disease‐target network by using Cytoscape 3.7.2 software (Figure 4). In the network diagram, purple represents Xingren, red represents COVID‐19, yellow represents the active ingredient of Xingren, and green represents their common genes. From the diagram, it can be seen that the action targets of different active ingredients can be the same or different, which reflects the multicomponent and multi‐target action mechanism of Xingren on COVID‐19, and the results are shown in Figure 4. More importantly, according to the degree values of active ingredients and key targets, the top four active ingredients were glycyrol, licochalcone B, glabridin, and phaseol, respectively. Meanwhile, the top four key targts were PTGS2, PPARG, MAPK14, and CCNA2, respectively.

### 3.4 GO functional enrichment analysis

**FIGURE 5:**
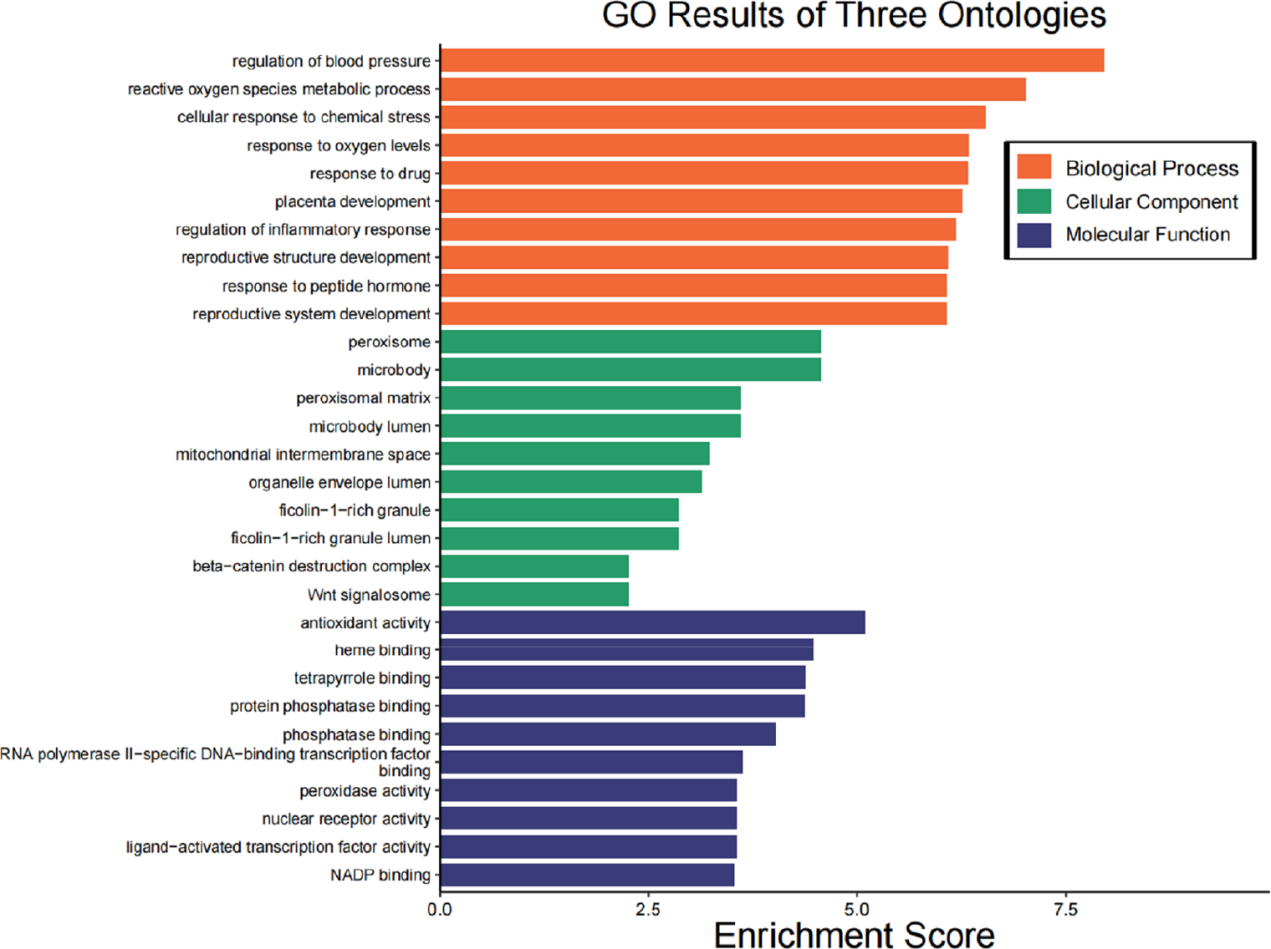
Results of GO functional enrichment functional analysis

Importing nine relevant key targets of Xingren into R 3.6.3 software and performing GO functional enrichment analysis by executing DOSE, clusterProfiler, and pathview in R 3.6.3 software and obtaining a total of 30 biological processes, molecular functions, and cellular components at *p* < .05, the results are shown in Figure 5. Among it, the common action targets are mainly enriched in regulation of blood pressure, reactive oxygen species metabolic process, cellular response to chemical stress, peroxisome, microbody, peroxisomal matrix, antioxidant activity, heme binding, tetrapyrrole binding, etc.

### 3.5 KEEG pathway enrichment analysis

**FIGURE 6:**
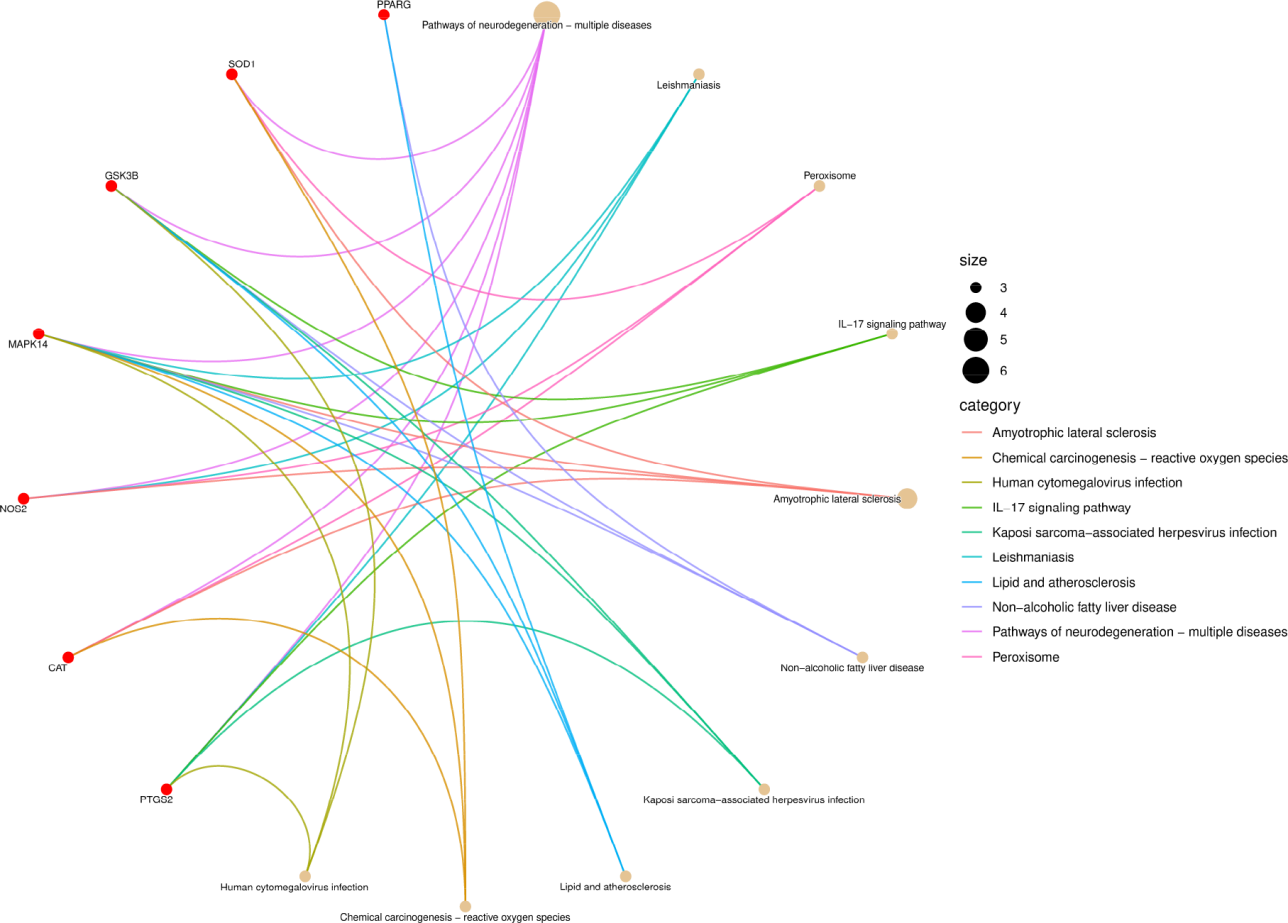
Pathway cnetplot of KEGG pathway enrichment analysis

**FIGURE 7:**
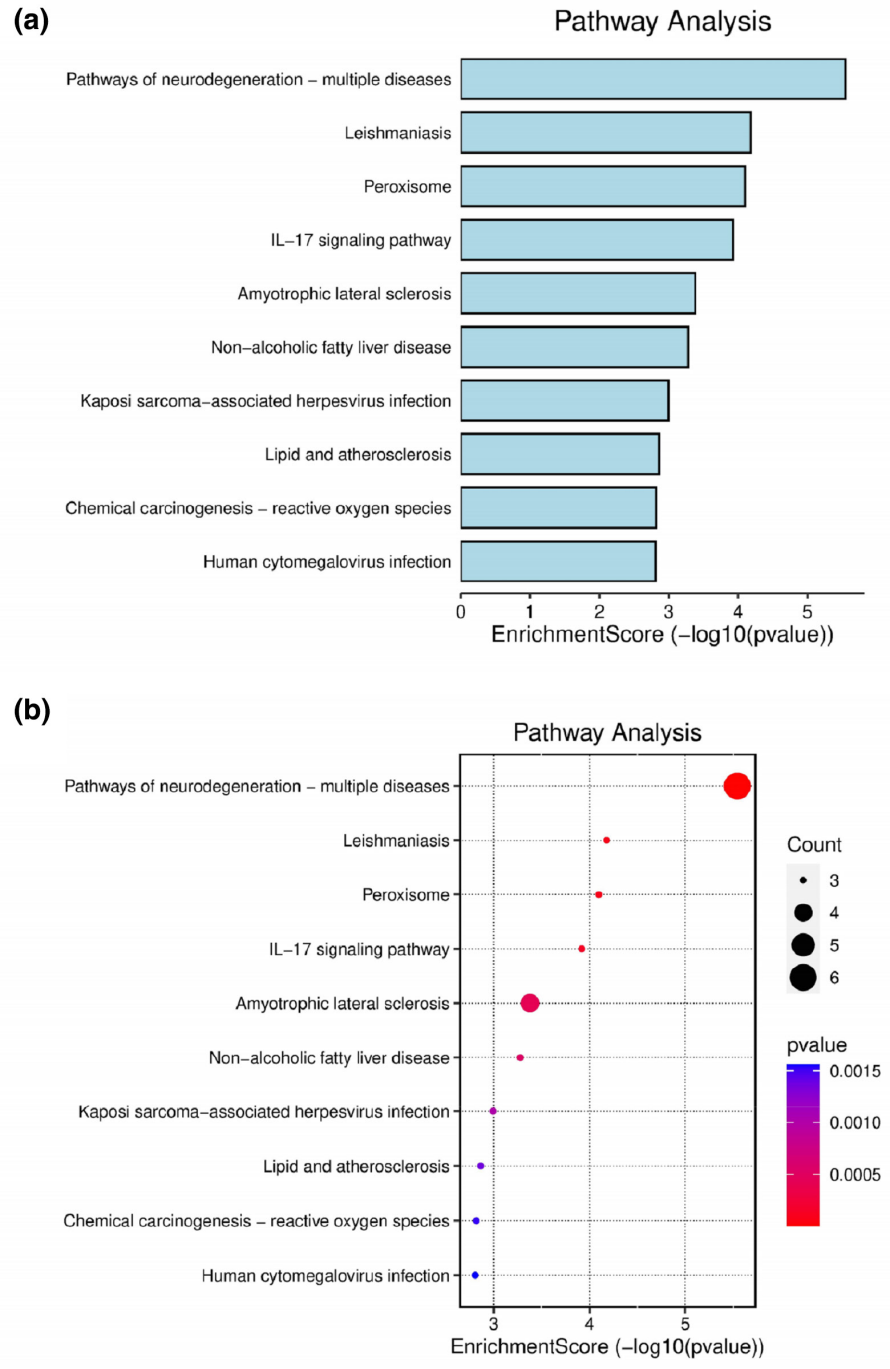
The results of KEGG pathway enrichment analysis. (a) Bar graph; (b) Bubble diagram

The nine related targets of almond were imported into R 3.6.3 software, and KEEG pathway enrichment analysis was performed by executing DOSE, clusterProfiler, and pathview in R 3.6.3 software, and *p* < .05 to a total of 52 signaling pathways. The findings presented the 10 signaling pathways with the smallest *p* values (Figures 6 and 7). Our results show that the key target genes are mainly in Pathways of neurodegeneration‐multiple diseases, Leishmaniasis Peroxisome, IL‐17 signaling pathway, Amyotrophic lateral sclerosis, Non‐alcoholic fatty liver disease, Kaposi sarcoma‐associated herpesvirus infection, Lipid and atherosclerosis, Chemical carcinogenesis‐reactive oxygen species, Human cytomegalovirus infection. In addition, VEGF signaling pathway, longevity regulating pathway‐multiple species, prolactin signaling pathway, longevity regulating pathway, C‐type lectin receptor signaling pathway, other signaling pathways, T‐cell receptor signaling pathway, and TNF signaling pathway are also involved, and the *p*‐value is less than .05.

### 3.6 Molecular docking

**FIGURE 8:**
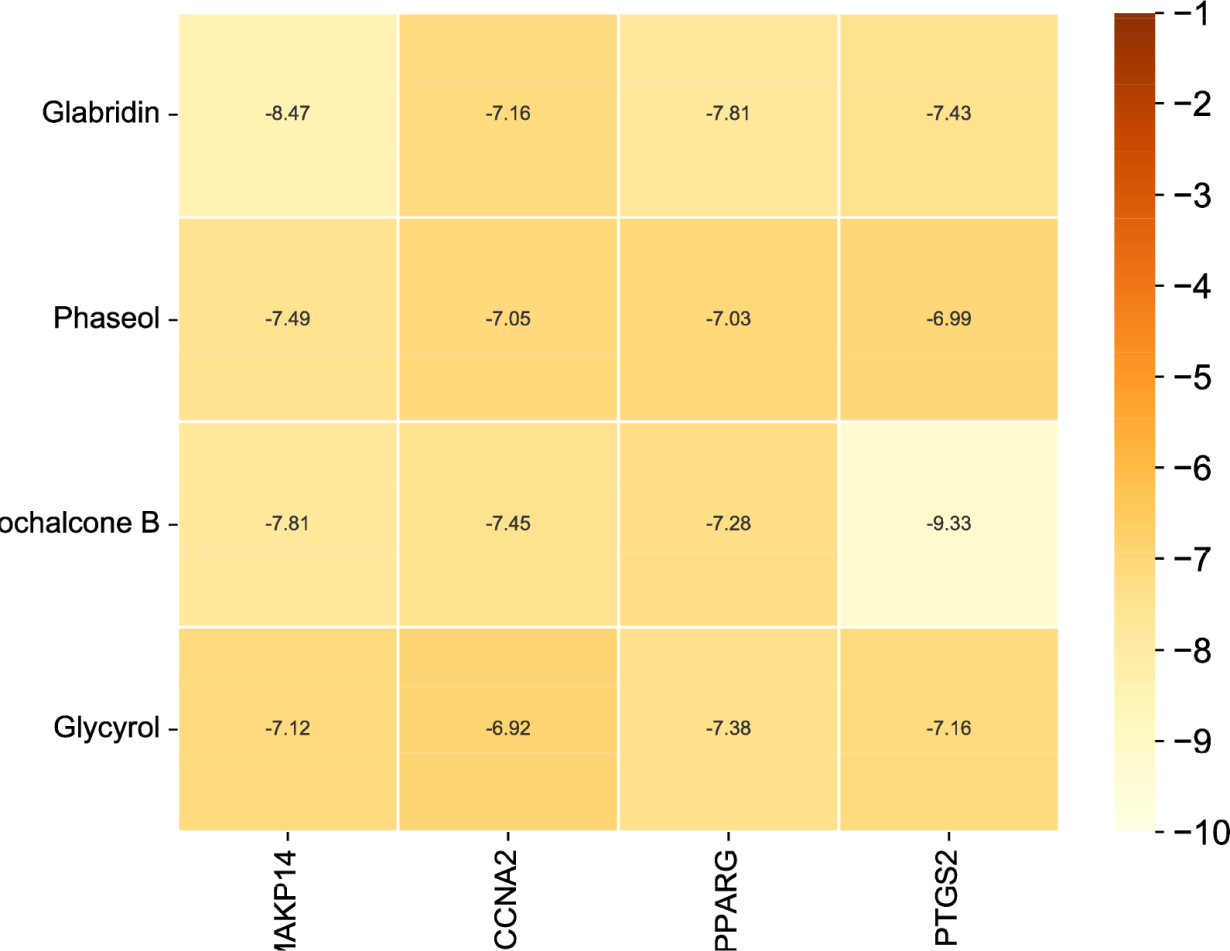
The heat map of molecular docking results

In this experiment, compounds glabridin, phaseol, licochalcone B, and glycyrol were molecularly docked with MAKP14, CCNA2, PPARG, and PTGS2 target proteins. The molecular docking results showed that the four compounds bind well to the four target proteins and have a good match, and the results are shown in Table 2. The complexes formed by the compound and the protein after docking were visualized using Pymol 2.1 software (the compound with the most negative docking score was selected for each target) to obtain the binding pattern of the compound to the protein, and the amino acid residues that bind the compound to the protein pocket can be clearly seen based on the binding pattern. For example, amino acid residues of licochalcone B that interact with the active site of PTGS2 protein include SER‐530, ARG‐120, TRP‐387, MET‐522, etc. Licochalcone B can form strong hydrophobic interactions with active site amino acids (TRP‐387, MET‐522), especially TRP‐387 can form π‐π conjugation interactions with the benzene ring of the compound, which is important for stabilizing small molecules in the protein cavity. In addition, this compound is able to form multiple hydrogen bond interactions with amino acids SER‐530 and ARG‐120, which make an important contribution to anchoring small molecules in the protein cavity. Several other compounds also have hydrogen bonding interactions and hydrophobic interactions with MAKP14, CCNA2, PPARG, PTGS2 targets, which can effectively promote the formation of stable complexes between small molecules and proteins. In summary, glabridin, phaseol, licochalcone B, and glycyrol showed good performance in docking scoring and binding mode with MAKP14, CCNA2, PPARG, PTGS2 target proteins, and were able to form stable complexes with the proteins with strong association with the four targets. The heat map based on the results of molecular docking is shown in Figure 8, which indicated that the lighter the color of the square, the closer the binding between the active ingredient and the target.

**TABLE 2:** Molecular docking results of the main four ingredients of Xingren with four core target proteins

Target	PDB ID	Target structure	Active ingredients	Affinity (kJ·mol^−1^)	Best‐docked complex (3D) and (2D)
MAKP14	6SFO	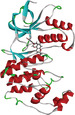	Glabridin	−8.47	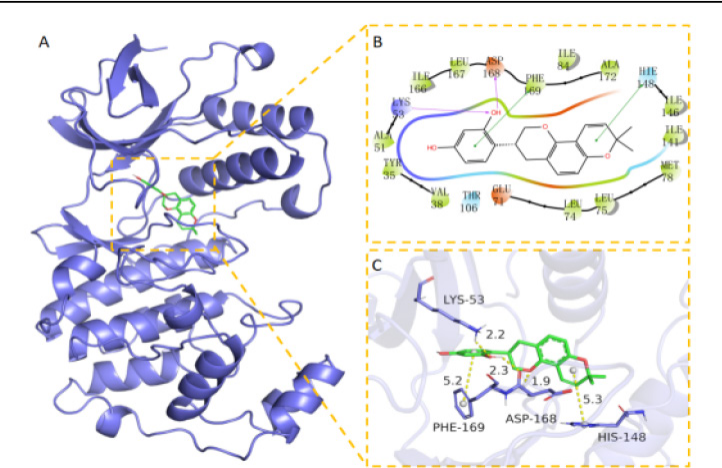
			Phaseol	−7.49	
			Licochalcone B	−7.81	
			Glycyrol	−7.12	
CCNA2	4EOJ	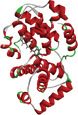	Glabridin	−7.16	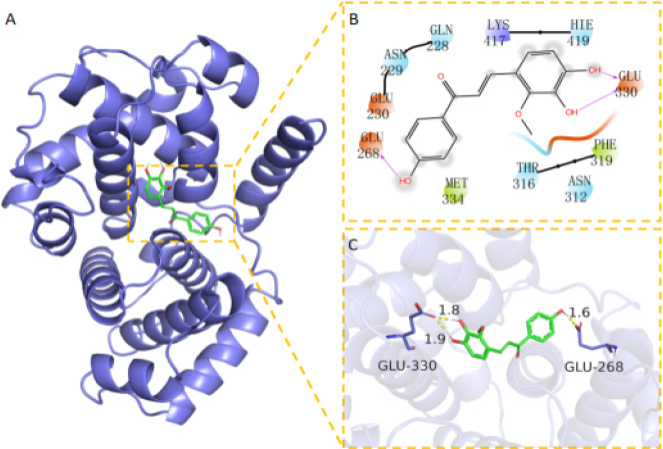
			Phaseol	−7.05	
			Licochalcone B	−7.45	
			Glycyrol	−6.92	
PPARG	7AWD	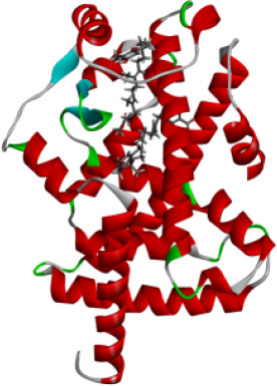	Glabridin	−7.81	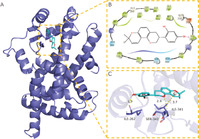
			Phaseol	−7.03	
			Licochalcone B	−7.28	
			Glycyrol	−7.38	
PTGS2	5IKR	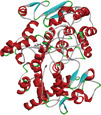	Glabridin	−7.43	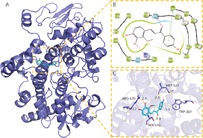
			Phaseol	−6.99	
			Licochalcone B	−9.33	
			Glycyrol	−7.16	

*Notes*: A is the 3D structure of the active ingredient in complex with the core target protein, B is the 2D binding mode of the complex, and C is the 3D binding mode of the complex.

## 4 DISCUSSION

In this study, the active ingredients of Xingren were first screened from the TCMSP platform. The GeneGards database was used to screen out the targets of COVID‐19, and the targets related to the active ingredient of Xingren were intersected with the targets related to COVID‐19, then construction of PPI networks and Xingren active ingredient‐disease‐target networks. We also performed GO function and KEGG pathway enrichment analysis of the active ingredient targets of Xingren using R 3.6.3 software, so as to discover the active ingredients of Xingren, the disease targets and the regulated pathways. The active ingredients of the top four Xingren in the Degree ranking were licochalcone B, phaseol, glabridin, and glycyrol.

Viral and bacterial infections cause inflammatory storms that accelerate disease progression, and this is also true for the COVID‐19 virus, which is a major factor in accelerating disease progression (Bird, 2018; Liu et al., 2016; Ye et al., 2020). According to the “Novel Coronavirus Pneumonia Treatment Protocol (Trial Version 9),” inflammatory exudates and mucus are seen in the lungs of patients with neocoronavirus infection, and inflammatory cells mainly include monocytes and lymphocytes (Fang et al., 2022). Neutrophil and lymphocyte infiltration is also seen in the heart and blood vessels, so that neocoronavirus can cause the production and release of inflammatory factors, while in a study by Xu it was also confirmed that the appearance of respiratory symptoms in patients with neocoronavirus infection is associated with the activation and release of inflammatory factors and endotoxins (Xu et al., 2020). Licochalcone B, a derivative of licochalcone, it can inhibit the inflammatory response in macrophages and to protect mice from endotoxic shock (Park et al., 2014). In an in vitro experimental study, licochalcone B inhibited NO and pro‐inflammatory cytokine production by suppressing the activation of nuclear factor‐kB and activator protein 1 in lipopolysaccharide in RAW264.7 cells. In animal models, licochalcone B may protect BALB/c mice from lipopolysaccharide‐induced endotoxic shock by inhibiting the production of inflammatory cytokines. Other researchers also found that licochalcone B has anti‐inflammatory, antibacterial, antioxidant and anticancer activities (Kang et al., 2017).

When Jiang and colleagues extracted active compounds from rhododendron clover root, its compound (phaseol) showed moderate inhibition and no cytotoxic effect when the anti‐inflammatory activity of phaseol was evaluated by inhibiting NO production in lipopolysaccharide‐activated murine macrophages RAW 264.7 cells (Jiang et al., 2019). Furthermore, in the p‐coumarin‐extracted phaseol by Li et al. was found to reduce LP26‐induced production of inflammatory mediators (e.g., NO, PGE2 and ROS) in RAW 264.7 macrophages (Li et al., 2017). The results of both suggest that phaseol may achieve anti‐inflammatory effects by inhibiting the production of inflammatory mediators.

In a study, Glabridin reduced inflammation and injury perception in rodents by activating BKCa channels and decreasing NO levels (Parlar et al., 2020). This compound shows an anti‐injury sensing response mainly by activating BKCa channels and downregulating NO levels and part of the transient receptor potential vanilloid‐1 pathway, it also shows anti‐inflammatory effects by inhibiting COX activity without cytotoxicity. Glabridin also reduces LDH activity and decreases LD concentration, thereby inhibiting glycolytic metabolism and regulating energy metabolism in breast cancer cells, and may be used as a potential anticancer agent or anticancer adjuvant (Li et al., 2019). One study has shown that Glabridin may also achieve therapeutic effects in atherosclerosis by regulating the expression and downregulation of the activity of MLCK (Wang et al., 2019). In addition, Glabridin has been suggested to have a regulatory effect on MAPK pathway signaling, but further studies are needed. In the opinions from Fu showed that Glycyrol inhibited IL‐2 expression by decreasing NF‐κB and NFAT transcriptional activity (Fu et al., 2014). It also has a therapeutic effect on autoimmune and inflammatory reactions, especially in rheumatoid arthritis diseases. Another study from Shin confirmed the anti‐inflammatory effect of Glycyrol, which was attributed to the inhibition of phosphorylation of I‐κBα (Shin et al., 2008).

In the active ingredient disease‐target network, a total of nine targets were obtained, namely PTGS2, CAT, NOS2, PPARG, MAPK14, GSK3B, CCNA2, AR, and SOD1. The top four of the degree rankings are PTGS2, PPARG, MAPK14, and CCNA2. PTGS2 are inflammatory response genes, and it is also closely related to the development of tumors. It has been found that PTGS2 play a key role in the development of ovarian and intestinal tumors (Cabrera et al., 2006; Chulada et al., 2000; Habermann et al., 2013; Yucesoy et al., 2016). At present, a number of studies have also confirmed that PTGS2 is a potential target for the treatment of COVID‐19, and it has great potential as a target for the development of new drugs (Li, Li, et al., 2021; Li, Qiu, et al., 2021; Passos et al., 2022). Two studies found that there are different receptors in the nuclear receptor family of PPARG, including PPARα, PPARβ, and PPARγ, among which PPARγ plays an important role in inflammation as well as in the immune system (Bandera Merchan et al., 2016; Toffoli et al., 2017). Besides, recent studies have highlighted the interesting role of PPARγ agonists as modulators of inflammatory and immunomodulatory drugs through the targeting of the cytokine storm in COVID‐19 patients (Vallée et al., 2019; Vallée & Lecarpentier, 2016, 2018). SARS‐CoV2 infection presents a decrease in the ACE2 associated with the upregulation of the WNT/β‐catenin pathway,and SARS‐Cov2 may invade human organs besides the lungs through the expression of ACE2 (Barbieri et al., 2020; Perrotta et al., 2020). Evidence has highlighted the fact that PPARγ agonists can increase ACE2 expression, suggesting a possible role for PPARγ agonists in the treatment of COVID‐19 (Vallée et al., 2021). MAKP14 is involved in cell differentiation, growth, proliferation, immune regulation and other processes. It has also been shown that when MAKP14 is suppressed, the chance of viral infection is reduced (Wang et al., 2017). Currently, no research report between MAKP14 and COVID‐19 has been found. CCNA2 is a regulator of cyclin‐dependent kinases, which exhibits the characteristics of changes in protein abundance with cell cycle during the cell cycle, and is a highly conserved member of the cyclin family (Gan et al., 2018). It has been found that CCNA2 expression is increased in many types of cancer (Jiang et al., 2022). Additionally, CCNA2 is also a potential therapeutic target for COVID‐19 infection caused by SARS‐CoV‐2 (Chen et al., 2022).

The enrichment analysis of GO function shows that the main enriched biological reaction entries are in regulation of blood pressure, reactive oxygen species metabolic process, cellular response to chemical stress, peroxisome, microbody, peroxisomal matrix, antioxidant activity, heme binding, tetrapyrrole binding, etc. These biological responses may have an extremely important role in Xingren against COVID‐19, where it has been shown that their responses may play a driving role in the occurrence and development of COVID‐19, such as cellular responses to chemical stress (Hightower & Santoro, 2020), antioxidant activity (Al‐Saleh et al., 2022), heme binding (Rapozzi et al., 2021), peroxisome (Donma & Donma, 2020), etc. KEGG pathway analysis showed that Xingren to COVID‐19 is involved in pathways of neurodegeneration‐multiple diseases, leishmaniasis peroxisome, IL‐17 signaling pathway, amyotrophic lateral sclerosis, Non‐alcoholic fatty liver disease, Kaposi sarcoma–associated herpesvirus infection, lipid and atherosclerosis, chemical carcinogenesis‐reactive oxygen species, and human cytomegalovirus infection. Analysis of the abovementioned pathways can involve multiple aspects such as inflammation, tumors, bacteria, and viruses. Of which the number of entries was more than five for pathways of neurodegeneration‐multiple diseases, and more than four for leishmaniasis and peroxisome. Among them, IL‐17 signaling pathway is an important pathway of action in inflammatory response, COVID‐19, tumors, etc. (Lv et al., 2022; Megna et al., 2020; Pan et al., 2022).

Additionally, the molecular docking results also showed that all four major active ingredients of Xingren bind well and match well with the four common target proteins between Xingren and COVID‐19. Among them, licochalcone B has a highest binding energy with PTGS2 (−9.33 kJ·mol^−1^). In this study, the active ingredient of Xingren, glabridin, also had better binding ability and good binding effect with MAKP14. To sum up, licochalcone B may also become a herbal monomer against COVID‐19, and it is hypothesized that it may inhibit the signaling flux of IL‐17, VEGF, prolactin, and C‐type lectin receptor pathway, by acting on multiple target proteins, thus achieving the effect against COVID‐19. For this reason, licochalcone B deserves further study and analysis.

## 5 CONCLUSION

In summary, Xingren have potential preventive and curative effects against COVID‐19. Although there are few common target proteins between the two, Xingren is active in many formulations targeting COVID‐19, and it is assumed that it is most likely to be used as a “minister” drug rather than a “ruler” drug, and its efficacy is good, which is of great value for research. In addition, we found that licochalcone B in Xingren binds well to PTGS2, the target protein of COVID‐19, and licochalcone B deserves further study and analysis. Finally, this study also provides some theoretical basis and reference for the excavation of the active ingredients of Xingren, licochalcone B to prevent and control COVID‐19 and the development of new drugs for COVID‐19.

## AUTHOR CONTRIBUTIONS

BY and MW designed the study; JW supervised the study; MW, YW, and LL performed the research, analyzed the data and wrote the manuscript; JW and BY revised the manuscript. All authors reviewed the manuscript. And all authors read and approved the final version of the manuscript.

## CONFLICT OF INTEREST

All authors declare that they have no conflicts of interest.

## Data Availability

The data that support the findings of this study are available from the corresponding author upon reasonable request.
